# The effect of the severity of liver cirrhosis on the level of lipids and lipoproteins

**DOI:** 10.1007/s10238-013-0262-5

**Published:** 2013-10-12

**Authors:** Lech Chrostek, Lukasz Supronowicz, Anatol Panasiuk, Bogdan Cylwik, Ewa Gruszewska, Robert Flisiak

**Affiliations:** 1 Department of Biochemical Diagnostics, Medical University of Bialystok, Waszyngtona 15A, 15-269 Bialystok, Poland; 2 Department of Infectious Diseases and Hepatology, Medical University of Bialystok, Zurawia 15, 15-540 Bialystok, Poland

**Keywords:** Lipids and lipoproteins, Severity of liver damage, Cirrhosis

## Abstract

The effect of severity of liver cirrhosis, an alcoholic and non-alcoholic genesis, on the results of serum lipids and lipoproteins was evaluated. Serum cholesterol, triglycerides, high-density lipoprotein cholesterol (HDL-Ch), and low-density lipoprotein cholesterol (LDL-Ch) were measured in the sera of 59 patients suffering from alcoholic cirrhosis and 34 patients with non-alcoholic cirrhosis. The level of serum triglycerides depends on the severity of liver damage in alcoholic liver cirrhosis, being the highest in Child-Pugh score B. The severity of liver damage significantly affects the HDL-Ch and LDL-Ch levels in cirrhosis of non-alcoholic origin, reaching the highest value for LDL-Ch and the lowest for HDL-Ch in score C. It should not be generalized that the levels of lipids and lipoproteins in liver cirrhosis progressively diminished with the deterioration of liver function. The serum HDL-Ch and LDL-Ch may be considered as markers of severity of liver damage in non-alcoholic cirrhosis, but the triglycerides only in disease of alcoholic origin.

## Introduction

The liver plays a crucial role in the synthesis, secretion, catabolism, and storage of lipids and lipoproteins. Therefore, the serum lipids and lipoproteins concentrations in liver diseases could be changed [1–4]. Generally, the level of plasma lipids and lipoproteins tends to decrease with the severity of liver disease [5, 6]. However, the results of these studies were not the same. The reason for the discrepancy of the results could be the different etiology of liver injury. There is no information if alcoholic and non-alcoholic liver cirrhosis equally affect the lipids metabolism. It has been known that the major effects of high alcohol consumption on lipids metabolism are the excessive synthesis of triglycerides, hypertriglyceridemia and hypercholesterolemia, defective esterification of plasma cholesterol, and decreased level of high-density lipoprotein cholesterol [7]. These changes should be contrasted with the effects produced by moderate alcohol consumption which can be reversible with cessation of alcohol drinking [8]. Therefore, the aim of this study was to evaluate the influence of liver cirrhosis severity, an alcoholic and non-alcoholic origin, on the concentration of serum lipids and lipoproteins.

## Materials and methods

Studies were carried out on 59 patients suffering from alcoholic cirrhosis (ALC) (14 females and 45 males) (mean age: 53 years; range: 28–78) and 34 patients with non-alcoholic cirrhosis (NALC) (16 females and 18 males) (mean age: 62 years; range: 20–84) who were admitted in the Department of Infectious Diseases and Hepatology. The alcoholic group consisted of alcohol-dependent patients. The diagnosis of dependency was made on the basis of ICD-10 criteria (World Health Organization, 1992). The self-reported mean alcohol consumption was 1,106 g of ethanol per week (range: 553–3,290), and mean time of dependency was 23 years (range: 10–44). The time after cessation of drinking was above 1 month. The following numbers of patients were in subgroups with ALC: 7 in score A, 21 in score B, and 26 in score C, and in subgroups with NALC: 11 patients in score A, 14 in score B, and 6 in score C. All patients with cirrhosis were tested for the prevalence of the hepatitis virus. The causes of non-alcoholic liver cirrhosis were HBV in 14 patients, HCV in 9 patients, and unidentified factors in the rest patients. Among unidentified cases, 2 patients had type 2 diabetes mellitus. Any patient was on previous drug treatment to lower cholesterol.

The diagnosis of cirrhosis was performed on the basis of signs and symptoms of the disease, physical and clinical examination (abdominal ultrasound and liver biopsy in selected cases), and biochemical liver panel known as liver function tests which included alanine and aspartate aminotransferase (ALT and AST), γ-glutamyl transferase (γ-GT), bilirubin, albumin, and total protein. The serological tests (HBsAg and anti-HCV) were also used to support the diagnosis of viral infections.

The control group consisted of 80 healthy subjects recruited from hospital workers (mean age 55 years; range 22–60; 36 females and 44 males). All subjects (healthy and sick) gave their consent to participate in the studies. Blood samples were taken fasting morning by vein puncture once after admittance, before treatment. The blood was allowed to clot. The sera were separated by centrifugation at 1,500×*g* for 10 min at room temperature and stored at −86 °C until analysis. This study was approved ethically by the Bioethical Committee working at the Medical University in Bialystok (Approval No. R-I-002/192/2009). All patients provided informed written consent.

Serum cholesterol (Ch), triglycerides (TG), high-density lipoprotein cholesterol (HDL-Ch) were measured by methods manufactured by Abbott Diagnostics (Wiesbaden, Germany) on the Architect c8000 analyzer (Abbott Laboratories, Abbott Park, USA). Low-density lipoprotein cholesterol (LDL-Ch) was calculated with Friedewald’s formula.

To test the effect of liver diseases on the concentration of lipids and lipoproteins, ANOVA rank Kruskal–Wallis test was performed. Because the chance of finding one or more significant differences in 3 tested groups was 14.26 % (Bonferroni correction factor), we performed the nonparametric multiple comparison test (post hoc test for Kruskal–Wallis) to ascertain which the intermediate medians are significantly different. The differences between tested and control groups were evaluated by Mann–Whitney *U* test. To calculate the correlation between variables, Spearman rank correlation coefficient was used. We considered *P* values less than 0.05 as statistically significant.

## Results

The concentration of lipids and lipoproteins are presented in Table 1. The concentration of cholesterol, HDL-cholesterol, and LDL-cholesterol in alcoholic and non-alcoholic cirrhosis were significantly decreased in comparison with the control group. The level of triglycerides was decreased in NALC and was lower than that in alcoholic ones.

**Table 1:** Serum lipids and lipoproteins in ALC and NALC

	Cholesterol (mmol/L)	Triglycerides (mmol/L)	HDL-cholesterol (mmol/L)	LDL-cholesterol (mmol/L)
Control group	4.98 ± 0.65	1.25 ± 0.37	1.32 ± 0.25	3.04 ± 0.54
Alcoholic cirrhosis	4.11 ± 1.67 *P* ^*k*^ < 0.001	1.47 ± 0.83 *P* ^*k*^ = 0.579	0.72 ± 0.48 *P* ^*k*^ < 0.001	2.72 ± 1.38 *P* ^*k*^ < 0.001
Non-alcoholic cirrhosis	3.68 ± 1.04 *P* ^*k*^ < 0.001 *P* ^*a*^ = 0.522	1.11 ± 0.51 *P* ^*k*^ = 0.017 *P* ^*a*^ = 0.017	0.89 ± 0.46 *P* ^*k*^ < 0.001 *P* ^*a*^ = 0.072	2.25 ± 0.78 *P* ^*k*^ < 0.001 *P* ^*a*^ = 0.085

Data are mean ± standard deviation. The differences between tested group and controls were estimated by Mann–Whitney U test, *P* ^*k*^ the differences between tested group and controls, *P* ^*a*^ the differences between tested groups

**Fig. 1:**
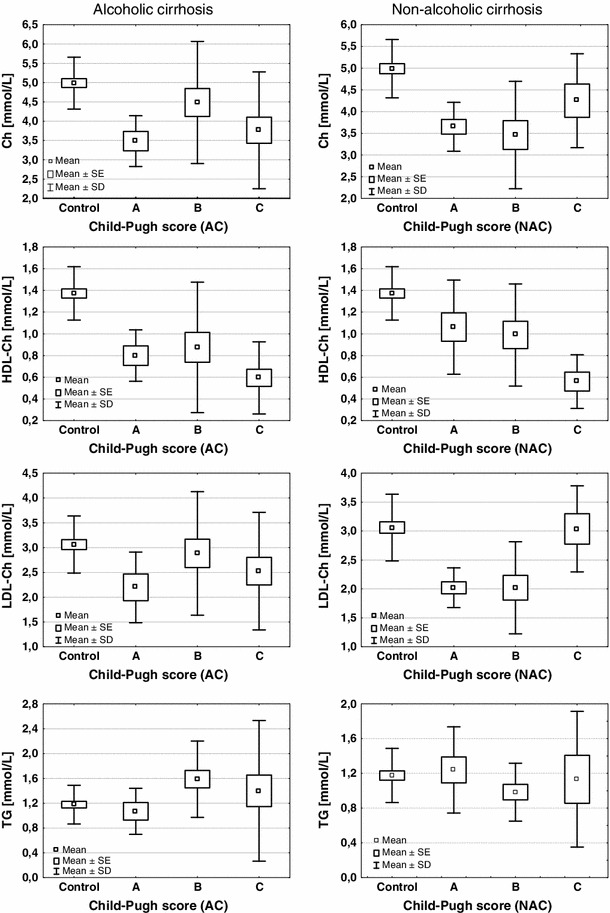
Serum concentrations of cholesterol (Ch), triglycerides (TG), high-density lipoprotein cholesterol (HDL-Ch) and low-density lipoprotein cholesterol (LDL-Ch) in liver cirrhosis. *Box* and *whiskers* plot illustrating the means, standard errors of mean (SE) and standard deviations (SD). A, B, C—Child-Pugh scores

In alcoholic liver cirrhosis, only the level of serum triglycerides appears to be dependent on the severity of liver damage evaluated by Child-Pugh score (*H* = 6.676, *P* = 0.036) (Fig. 1). The mean TG level in score B (1.58 ± 0.61 mmol/L) was significantly higher than that in score A (1.07 ± 0.37 mmol/L; *P* = 0.049) and score C (1.40 ± 1.13 mmol/L; *P* = 0.026). In NALC, both, LDL-cholesterol and HDL-cholesterol, significantly depend on the degree of liver damage (*H* = 9.660, *P* = 0.008 for LDL-cholesterol and *H* = 7.641, *P* = 0.022 for HDL-cholesterol) (Fig. 1). Further analysis (post hoc) revealed the significant differences between score A (2.02 ± 0.34 mmol/L) and score C (3.04 ± 0.74 mmol/L) (*P* = 0.034) and between score B (2.02 ± 0.79 mmol/L) and score C (*P* = 0.009) for LDL-cholesterol and between score A (1.06 ± 0.43 mmol/L) and score C (0.56 ± 0.25 mmol/L) (*P* = 0.029) for HDL-cholesterol. The additional comparison between cases with viral and non-viral etiology of disease was made in NALC. Two cases of diabetes were excluded from the evaluation. These comparisons revealed a statistical significant differences between viral and non-viral etiology of NALC only for LDL-cholesterol (1.77 ± 0.50 mmol/L for viral vs. 2.45 ± 0.79 mmol/L for non-viral cases; *P* = 0.011). Contrary to the above result, the glucose concentration in the non-viral group (6.42 ± 2.76 mmol/L) was significantly higher (*P* = 0.017) than that in the viral group (4.97 ± 0.91 mmol/L). The correlation study showed that only HDL-cholesterol correlated with the severity of liver disease in both, ALC and NALC (*R* = −0.407, *P* = 0.005 and *R* = −0.464, *P* = 0.008, respectively).

## Discussion

In general, the plasma lipids and lipoproteins tend to decrease with parenchymal liver disease, and the level and composition of the lipoproteins depends on the activity of enzymes involved in lipid metabolism. These include lipoprotein lipase (LPL), lecithin-cholesterol acyltransferase (LCAT), and hepatic triglyceride lipase (HTGL) [4, 9]. The results of this study confirm that the concentrations of lipids and lipoproteins in the liver diseases are changed [1–4]. The mean concentrations of cholesterol, HDL-cholesterol, and LDL-cholesterol were significantly decreased in liver cirrhosis of both origin (alcoholic and non-alcoholic). The triglycerides level diminished only in NALC and was lower than that in alcoholic ones. In circulation, the triglycerides-rich lipoproteins comprise very low-density lipoprotein (VLDL) and chylomicrons. The assembly and secretion of VLDL particles take place in the liver cells and both elements, apolipoprotein B and microsomal triglyceride transfer protein (MTP), are necessary for these processes [10]. It has been shown that MTP plays a role in transferring lipid to nascent apolipoprotein B and hepatic induction of MTP, resulting in a reduction in hepatic TG accumulation and improvement of VLDL export, which increases the serum level of TG [11]. The reason of that may be the involvement of MTP in the import of triglycerides into the lumen of endoplasmic reticulum.

The evidence showed that cholesterol and its fractions progressively decrease with increasing severity of the disease, however, without differentiation in the origin of illness [2]. In our study, the fractions of cholesterol differ according to etiology of liver cirrhosis. In non-alcoholic liver cirrhosis, HDL-cholesterol concentration diminished with the severity of disease, being lower in severe liver damage (score C) than that in mild damage (score A). In contrast, LDL-cholesterol level in severe liver damaged (score C) was higher than that in less damaged organ (score A and B). In ALC, only the level of triglycerides changes with the severity of liver damage (according to the analysis of variance), although, the level of TG was not highest in score C, but in score B. Class C is considered as decompensated disease (with severe ascites and encephalopathy), in which the ability of the liver to synthesize and β-oxidation of fatty acids, and to synthesis of TG, is reduced. Ramcharran and co-workers [12] have found that more severe liver disease is associated with lower lipid levels, with the exception of TG levels that is directly related to steatosis. Therefore, it could not be generalized that the levels of lipids and lipoproteins in liver cirrhosis progressively diminished with the deterioration of liver status. The decreased HDL-cholesterol and LDL-cholesterol levels in liver cirrhosis might be explained by the decreased synthesis of apolipoproteins A and B [13]. The level of apolipoprotein A-1 was found to be low in cirrhosis, and its value was higher in Child-Pugh A group than that in Child-Pugh B and C groups. Therefore, apolipoprotein A-1 was recommended the most affected lipid parameters in liver injury among lipids [6]. Habib et al. [14] proposed that decline in lipoprotein cholesterol may reflect deterioration in liver function and of the lipoproteins, the HDL-cholesterol is the best liver function test and predictor of survival in cirrhotic patients.

Many data showed that chronic liver diseases strongly impair the lipid metabolism with the hypocholesterolemia as a common finding in cirrhosis and viral hepatitis [15, 16]. Additionally, the lower level of serum lipids is associated with more severe disease. In patients with chronic hepatitis C, lipid parameters were significantly lower in those with fibrosis than in those without fibrosis [12], and grading score was positively correlated with total cholesterol and LDL-cholesterol [17]. In patients with HCV, the lipogenesis is elevated while cholesterol synthesis is impaired [18]. Viral liver cirrhosis (HCV and HBV) also dysregulated the host lipid metabolism. However, there were many differences in lipid values between HCV and HBV cirrhosis [16]. In patients with cirrhosis, total serum cholesterol, LDL, and HDL-cholesterol were lower than that in patients with chronic active hepatitis [2]. Our study revealed that LDL-cholesterol was lower in viral etiology of cirrhosis than that in non-viral. We can speculate that the non-viral etiology of cirrhosis may be the metabolic syndrome. For this purpose, we tested the glucose level and denoted that its level in non-viral group was higher than that in viral one. This would confirm the metabolic etiology of non-viral cirrhosis, it means insulin resistance, which can develop into non-alcoholic steatohepatitis (NASH).

We conclude that it should not be generalized that the levels of lipids and lipoproteins in liver cirrhosis progressively diminished with the deterioration of liver function. On the basis of our results, the serum HDL-cholesterol and LDL-cholesterol may be considered as markers of the severity of liver damage only in NALC, but the triglycerides in cirrhosis of alcoholic origin.
